# Using Vertebrate Stem and Progenitor Cells for Cellular Agriculture, State-of-the-Art, Challenges, and Future Perspectives

**DOI:** 10.3390/biom12050699

**Published:** 2022-05-13

**Authors:** Teodora Knežić, Ljiljana Janjušević, Mila Djisalov, Supansa Yodmuang, Ivana Gadjanski

**Affiliations:** 1 Center for Biosystems, BioSense Institute, University of Novi Sad, Dr. Zorana Djindjica 1, 21000 Novi Sad, Serbia; teodora.knezic@biosense.rs (T.K.); ljiljana.janjusevic@biosense.rs (L.J.); mila.djisalov@biosense.rs (M.D.); 2 Research Affairs, Faculty of Medicine, Chulalongkorn University, 1873 Rama 4 Rd, Pathumwan, Bangkok 10330, Thailand; supansa.y@chula.ac.th

**Keywords:** cellular agriculture, stem cells, progenitor cells, tissue engineering, cultured meat, cultured seafood

## Abstract

Global food systems are under significant pressure to provide enough food, particularly protein-rich foods whose demand is on the rise in times of crisis and inflation, as presently existing due to post-COVID-19 pandemic effects and ongoing conflict in Ukraine and resulting in looming food insecurity, according to FAO. Cultivated meat (CM) and cultivated seafood (CS) are protein-rich alternatives for traditional meat and fish that are obtained via cellular agriculture (CA) i.e., tissue engineering for food applications. Stem and progenitor cells are the building blocks and starting point for any CA bioprocess. This review presents CA-relevant vertebrate cell types and procedures needed for their myogenic and adipogenic differentiation since muscle and fat tissue are the primary target tissues for CM/CS production. The review also describes existing challenges, such as a need for immortalized cell lines, or physical and biochemical parameters needed for enhanced meat/fat culture efficiency and ways to address them.

## 1. Introduction

It is a well-known fact that the constant rise of the human population, which is estimated to reach over 10 billion by the year 2100 [1] puts enormous pressure on the global food systems, particularly concerning the protein-rich foods, such as meat and seafood. In the current state of the world in the year 2022, with the still ongoing COVID-19 pandemic [2,3] and monumental political and economical changes in the global landscape associated with the conflict in Ukraine [4] that may even trigger serious food insecurity in Ukraine and whole Europe, according to Food and Agriculture Organization of the United Nations (FAO) [5], an important aspect to keep in mind is a confirmed observation that in a crisis, in inflation, there is increased demand of protein-rich foods [6]. In fact, FAO predicts a global shortage of protein-rich foods in the years to follow [7]. 

In addition, increased food production needs to be realized sustainably, not affecting already ongoing climate changes caused by agriculture-related factors such as industrial farming nor perpetuating the ongoing decline in marine/river wildlife populations due to overfishing [8]. 

**Alternative proteins (AP)** offer good new options for addressing rising global protein-rich food demand as they have the potential to significantly reduce food system emissions, free up significant amounts of land for additional climate mitigation strategies, food security, as well as for the protection of biodiversity while, in the same time securing sufficient amounts of protein-rich foods [9]. 

There are various categories of AP, and many are already being commercialized such as plant-, algae-, fungi-, insect-based AP, etc. [10], while the AP obtained by **cellular agriculture (CA)** still face a number of challenges related to scaling up and widespread commercialization [11]. 

In this review, we focus on **cultured meat (CM), cultured fat (CF),** and **cultured seafood (CS)** i.e., protein-rich alternatives for traditional meat and fish that are obtained via CA i.e., tissue engineering (TE) applied for food production. 

More precisely, we emphasize vertebrate cell types and procedures needed for myogenic and adipogenic differentiation, since muscle and fat tissue are the primary target tissues for CM/CF and CS production. This implies that under CS, within the scope of this review, we will be considering only fish and not crustaceans (shrimp, crab, lobster), mostly due to the scarcity of publicly-available information concerning crustacean-based CS, which is a field where Singaporean company Shiok Meats leads the global efforts.

As this review focuses on the cell-related aspects of CA-procedures for obtaining CM/CF/CS products, we invite the reader to consult other excellent recent reviews and publications that cover other aspects relevant to CA, such as support structures for cell immobilization comprising microcarriers (MCs) [12,13] and 3D scaffolds [14,15,16,17,18], types of bioreactors [19,20,21,22], bioprocess monitoring options [19,20,23] and methods of fabrication that can be utilized in CA [15,24,25], as these will not be covered in the current review in detail. In addition, we ought to mention two recent reviews that deal with the topics of cell types of relevance for CM/CS, but with a different focus than the current review. Shaikh et al. provide a short overview of various cell types for use in CA and provide extensive discussion on the biological effects of various myokines and cytokines on skeletal muscle and myogenesis [26], while Reiss et al. provide ample details on the bioprocessing aspects in relation to different cell types for CM [27]. 

In the current review, we opted to focus more on the topics that were not covered in mentioned reviews, hence we provide a detailed overview of the co-culture of myoblasts and adipocytes as well as different ways to stimulate differentiation towards myogenic and adipogenic lineages. 

**Figure 1:**
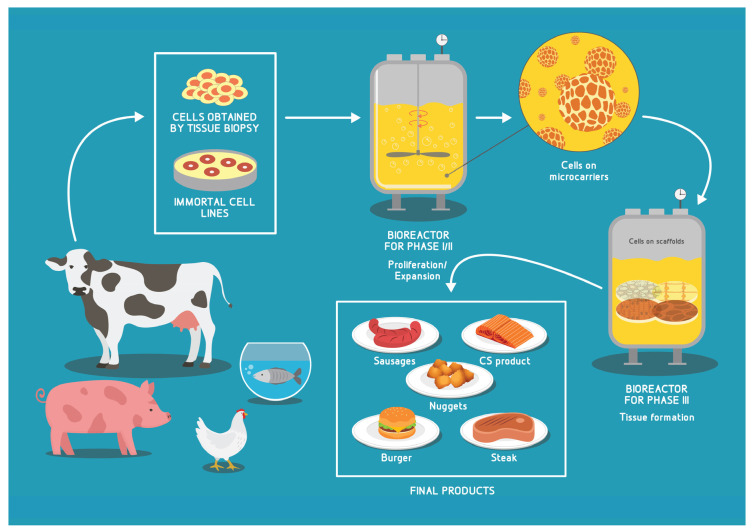
Simplified schematic of the vertebrate cell-based CM/CS bioprocess.

For a better understanding of the general concept of CA-based CM/CS bioprocess, we summarize in Figure 1 the main steps. 

In general, a CM/CF/CS bioprocess can be divided into four phases. Phase I involves cell isolation/acquirement and initial cell proliferation, in order to increase the cell numbers prior to initiating differentiation. The procurement of cells can occur from primary sources such as native animal tissue. In this context, we refer the readers to the excellent recent review by Guan et al. [28] where the authors discuss several methods of isolation and purification of muscle stem cells, which are the main cell type of interest for CM. The other option is to utilize immortal cell lines. Since vertebrate cells are, in major part, anchor-dependent, in order to enable a suspension culture system in dedicated bioreactors, it is necessary to seed the cells on MCs—structures with high surface-to-volume ratio, that provide support for adherent cell growth and expansion [12]. 

Phase II involves further cell expansion on a large scale, while Phase III comprises differentiation or muscle/fat tissue formation (maturation) on 3D scaffolds which leads to the final Phase IV comprising processing into final food products, either unstructured (such as burgers, sausages, nuggets) or structured such as “whole-cut”-like steaks and CS fillets [29]. 

## 2. Stem Cell and Progenitor Types Relevant in Cultured Meat (Mammalian, Avian) and Cultured Seafood (Fish) Bioprocesses

The discovery of stem cells and progenitor cells as their descendants, created better opportunities for in vitro production of CM/CF and CS, i.e., in vitro myogenesis and adipogenesis. This process involves stem or progenitor cell sampling, which can be done by performing a biopsy on living donor animals, ex vivo multiplication of the isolated cells, and TE techniques. It is important to emphasize that industrial-scale CM/CS production relies heavily on the process of cell proliferation and expansion, which is in line with stem cell multiplication properties.

For the CM/CF/CS production bioprocess, the following stem and progenitor cell types are of interest: pluripotent stem cells—embryonic stem cells (ESCs) and induced pluripotent stem cells (iPSCs); adult stem cells (ASCs)—mesenchymal stem cells (MSCs), adipose tissue-derived stem cells (ADSCs) and fibro-adipogenic progenitors (FAP), as well as resident muscle stem cells/muscle satellite cells (SCs), a.k.a. myosatellites and myoblasts as progenitors or more precisely, proliferating activated SCs. 

Depending on the isolated stem or progenitor cell type, these cells can differentiate into myocytes (muscle cells), adipocytes (fat cells), chondrocytes, or fibroblasts. 

### 2.1. Myogenic Differentiation–Satellite Cells (SCs) and Myoblasts

**Muscle satellite cells (SCs)** were first described in 1961 by Alexander Mauro [30]. These multipotent, adult, muscle-derived stem cells located on the periphery of myofibrils—between the basal lamina and sarcolemma, play a crucial role in the development and regeneration of skeletal muscle tissue [31]. They are mostly dormant cells, however, in order to regenerate muscle tissue after injury, they transform into actively-proliferative myoblasts—SC-amplifying progeny. Mononucleated myoblasts further differentiate and form myotubes, which will be packed into muscle fibers—myofibers [27,31]. At the same time, SCs are multiplying, which increases their number for future needs. When it comes to the genetic level, during the onset of their differentiation into skeletal myoblasts, SCs begin to express the transcription factor myoblast determination protein 1 (MyoD). The *MyoD* gene belongs to the early myogenic regulatory factor (MRF) genes, along with *Myf5*. These genes initiate cell expansion and are crucial for skeletal muscle to be properly formed [32]. The other group of MRF genes, *MRF4* and *Myogenin*, initiate cell differentiation and fusion. These genes are significant markers for cell monitoring and optimizing the composition of the culture medium for CM/CS production. 

In this regard, due to their easy and efficient differentiation, SCs have been selected as the most promising cell type for initiating CM/CS production. 

#### 2.1.1. Mammalian Myogenic Cells 

**Satellite cells (SCs)** have been initially successfully isolated from bovine carcasses and fetuses [33] and later standardized protocols for muscle biopsy from live animals were developed. As one of the cell types relevant for CM production, bovine SCs obtained by muscle biopsy were used to produce the world’s first CM prototype (lab-grown burger), and more recently to form contractile 3D bovine muscle tissue for the construction of millimeter-thick cultivated steak [34]. Also, the growth rate of bovine myoblasts in bioreactors for the CM production bioprocess was investigated [35]. For sustainable CM production, Okamoto et al. in their study showed the beneficial effects of nutrients extracted from the photosynthetic autotrophic microalgae *Chlorella vulgaris* on the proliferation and differentiation of primary bovine myoblasts [36]. Also, Haraguchi and Shimizu recently published a paper on the fabrication technique of 3D tissue using co-cultivation of *C. vulgaris* and animal cells for the production of healthy and thicker cultured food [37]. In addition to skeletal muscle cells, bovine aortic smooth muscle cells in edible gelatin fiber scaffolds were also cultivated in order to produce CM [38]. 

Muscle SCs have also been successfully isolated and differentiated from other agriculturally important species such as pigs [33,39,40], horses [40,41], and rabbits [38]. Besides cattle, there are also published studies on other ruminants such as sheep [42] and goats [43,44]. In this regard, Yamanouchi et al. conducted the first study on SC differentiation in goat skeletal muscle single fiber culture as an in vitro model [44]. Also, it has been shown that goat SCs are multipotent and can differentiate into myoblasts and adipocytes [45].

#### 2.1.2. Avian Myogenic Cells

Successful isolation of SCs also applies to avian species such as chicken [40,46], duck [40], and turkey [47]. In this regard, Nihashi et al. in their study presented effective isolation, proliferation, and differentiation of myoblasts from layer and broiler chicken [48]. In terms of dietary use, Eat Just, Inc. company from the US, produces in vitro cultivated “chicken nuggets” that are made by growing chicken cells in bioreactors and combining them with plant-based ingredients. This product is served in restaurants in Singapore, given that in December 2020, the Singapore Food Agency (SFA) approved the consumption of CM products [49]. Another US company, UPSIDE Foods (formerly Memphis Meats) also produces chicken and duck meat.

#### 2.1.3. Fish Myogenic Cells

When it comes to fish, Powell et al. proved already in the 90-ties of the XX century that SCs and myoblasts can be efficiently isolated from the rainbow trout (*Salmo gairdneri)* muscle and differentiated in vitro into myotubes [50]. Koumans et al. developed a method for isolating and purifying SCs from the white epaxial muscle of *Cyprinus carpio* and concluded that the in vitro behavior of SCs isolated from carp differs from that described for mammalian and avian SCs [51]. in vitro cultures of Atlantic salmon (*Salmo salar* L.), channel catfish (*Ictalurus punctatus*) and gilthead sea bream (*Sparus aurata*) SCs have also been successfully established. Also, an efficient protocol for isolation and in vitro maintenance of SCs and myoblasts has been optimized by Froehlich et al. and can be applied to Danioninae, as well as to rainbow trout, salmon, sea bream, etc. [52].

In addition, when it comes to dietary use, the National Aeronautics and Space Administration (NASA) has supported the first research aimed at in vitro production of edible muscle protein from *Carassius auratus* (goldfish) for astronauts [53]. Today, there are many companies engaged in CS production, such as California-based BlueNalu, which produces cell-cultured tuna, mahi-mahi, and red snapper as well as the first CS company in Europe—Berlin-based Bluu Seafood, which engages in the production of cultivated salmon, trout and carp.

However, having published studies on efficient isolation and in vitro maintenance of a certain cell type does not automatically imply the authors continued with the efforts toward CM/CS production. In the majority of cases, the main result of the study was the isolation protocol itself, and the characterization of obtained cells. What is largely missing from the majority of referenced studies is the next step—utilizing isolated cells in 3D culture, with support structures, aiming to engineer a tissue construct.

#### 2.1.4. Maintaining SCs Stemness through p38 Pathway

Improving the proliferative capacity of ASCs is an effective approach for scaling-up up specific cell sources, such as SCs, which is very important for developing the CM production bioprocess. Thus, scaling-up of SCs can be realized by keeping these cells in a proliferative, non-differentiated state, where this effect can be extended by in vitro inhibition of the p38 mitogen-activated protein kinase (MAPK) cell signaling pathway [54]. In this regard, Ding et al. demonstrated that the p38α/β inhibitor SB203580 inhibited the bovine SCs differentiation in short-term experiments, while long-term in vitro cultivation with p38i helped maintain the stemness and differentiation capacities [33]. Additionally, long-term cultivation with a p38 inhibitor also contributed to the maintenance of pig muscle stem cells [55].

When it comes to the avian species, there are indications that gga-miR-3525 regulates the proliferation and differentiation of SCs by targeting PDLIM3 via the p38/MAPK signaling pathway in chickens [56]. Also, PDLIM5 has a positive effect on chicken SC proliferation and differentiation via this pathway [57].

### 2.2. Embryonic Stem Cells (ESCs)

ESCs are suitable for cultivation because they multiply easily and are pluripotent i.e., can differentiate into any type of cell. They originate from the inner cell mass of embryos at the blastocyst phase. ESCs have been successfully isolated from a variety of organisms including humans, mice, chickens [58], fish [59], and cows [60]. ESCs can also be obtained from embryos formed by in vitro fertilization which is one of the options for acquiring pig ESCs [61] which are otherwise difficult to isolate, as discussed below.

Although ESCs can multiply indefinitely and differentiate into any type of cell, this process is quite expensive because it requires specialized media and controlled conditions for maturation into the desired tissue types [62,63]. Furthermore, the isolation of these cells implies the destruction of the embryo, which is an ethical problem. In addition, for some species, like pigs, the well-characterized ESCs lines have not been established. For both challenges, iPSCs are viable alternatives, as described in Section 2.3. Nevertheless, in the past decade, there has been significant progress in the establishment of livestock ESCs lines [64].

#### 2.2.1. Mammalian ESCs

Most research up to date has been performed on human/primate and murine ESCs, however, since neither are relevant for CA, we will focus on other mammalian ESCs that may be of relevance for CM production, such as cattle and sheep.

Attempts to derive stable bovine ESCs line have been ongoing for years, and have only recently been successful [60]. However, the bovine ESCs still require a custom-made medium as well as a mouse embryonic fibroblast (MEF) feeder layer. Recently, Soto et al. reported a simplified feeder-free culture protocol where they use commercially available medium and vitronectin substrate with Activin A supplementation which eliminates the need for the feeder layer. This protocol yields bovine ESCs that are stable in long-term culture i.e., express pluripotency markers and actively proliferate for more than 35 passages while keeping normal karyotype [65].

Similar to other livestock ESCs, until recently there was no established stable sheep ESC line. In 2020, Vilarino et al. reported the derivation of sheep ESCs under a chemically defined culture system containing fibroblast growth factor-2 (FGF-2) and a tankyrase/Wnt inhibitor (IWR1), yielding cells that maintain an euploid karyotype and stable expression of pluripotency markers after more than 40 passages [66].

#### 2.2.2. Avian ESCs

Embryonic cells for the production of poultry meat can be obtained from eggs. In chickens, the egg is laid 20 to 23 h after fertilization. Early embryonic development is divided into 14 phases (I-XIV) [67]. The fertilized cell goes through a fast phase of division and is laid in the embryonic stage X consisting of 20,000 to 50,000 blastodermal cells.

Even though chicken ESC isolated from eggs in the X phase have great potential for biotechnological applications, their long-term maintenance has not yet been fully achieved. In their recent review, Xiong et al. provide a good summary of conditions and parameters that need to be considered and optimized in order to maintain chicken ESCs pluripotency in the long-term culture [68]. The authors conclude that serum-free/feeder-free chicken ESCs culture conditions are more desirable in order to realize a stable, long-term chicken ESC line [68].

#### 2.2.3. Fish ESCs

Concerning fish ESCs, there is a number of research studies devoted to the generation of haploid ESCs as they are a useful model for analyzing mutations in recessive gene alleles which would not be apparent in the heterozygous animals. Fish models (mainly zebrafish—*Danio rerio* and medaka fish—*Oryzias latipes*) are good for generating haploid ESCs since a fertile adult can be produced after the nuclear transfer of a haploid ESC into a normal egg [69]. However, for CS purposes, diploid fish ESCs lines are of more relevance, as the ESCs need to be differentiated into muscle and fat tissues. Hong et al. established feeder-free conditions for ESCs derived from mid-blastula embryos of the medaka fish and further obtained several stable cell lines that show all features of in vitro mouse ESC line [70]. One of these lines, MES1, has been demonstrated to maintain a diploid karyotype in long-term culture (over 1 year of culture with more than 100 passages) and can be induced in vitro to differentiate into various cell types, including muscle cells [71].

### 2.3. Induced Pluripotent Stem Cells (iPSCs)

In addition to SCs, which are the most commonly used cell type for CM production, pluripotent stem cells also have great potential for utilization. Pluripotent stem cells, such as iPSCs, have a high proliferation capacity in vitro and, unlike SCs, iPSCs have an unlimited renewal capacity and the ability to differentiate into any cell type present in meat. iPSCs are produced by reprogramming somatic cells into an embryonic-like pluripotent state by inducing the expression of pluripotency-associated genes that encode transcription factors such as KLF4, SOX2, and MYC, NANOG, and POU5F1 (OCT4) [27,72,73]. However, these transcription factors may be species-dependent, e.g., in addition to POU5F1 and NANOG, bovine iPSCs also express stage-specific embryonic antigens (SSEAs)—SSEA1, SSEA3, and SSEA4, which are not expressed in human pluripotent stem cells [74]. This factor-based species difference can be extremely important for pluripotent cell line development from livestock species relevant for CM production.

#### 2.3.1. Mammalian iPSCs

Although the process of establishing pluripotent stem cell lines from relevant species such as cow [75], pig [55,76], goat [77], horse [78], and sheep [66,79] has progressed, it is still not at the large-scale level that is needed for the CM production bioprocess. However, there are promising studies such as those by Amilon et al. in which they generated functional skeletal myotubes from equine iPSCs [80,81]. Also, Genovese et al. have presented in their study a method for efficient in vitro creation of skeletal muscle from porcine iPSCs, with potential application in CM production [82].

#### 2.3.2. Avian iPSCs

When it comes to other agriculturally important species such as avian species, it appears to be far more complicated to achieve somatic cell reprogramming with them. Attempts to generate quail, chicken, or zebra finch iPSCs using mammalian reprogramming factors have resulted in the creation of partially reprogrammed cells [83,84]. However, for the first time, Yu et al. generated chicken iPSCs from fibroblasts using a nonviral minicircle reprogramming technique [85]. Also, Kim et al. generated iPS-like cells from avian feather follicular cells (FFCs) using retroviral vectors and suggested that FFCs are an alternative cell source for chicken cell reprogramming into iPSCs [86].

#### 2.3.3. Fish iPSCs

As for the fish iPSCs, Peng et al. published the technology for generating stable iPS-like cell lines from adult zebrafish (*Danio rerio*) fibroblasts, which, due to easy maintenance, can be applied to research other fish genera [87]. Also, Xu et al. recently published a study in which iPS-like cells were for the first time generated from koi fish (*C. carpio haematopterus*) caudal fin fibroblasts with a pure chemical reprogramming technique. This method is a promising strategy that can be applied to more fish species [88].

It is important to emphasize that several approaches have recently been proposed to create iPSCs without any genetic modifications such as transfection, which is a promising strategy for CM production using iPSCs because it may increase customer acceptance, due to the general aversion to “GMO/bio-engineered products” present in the general public [89,90].

From the industry-relevant side, an interesting example is a technology used by Dutch CM company Meatable. The company licensed a proprietary technology called OPTi-OX (optimized inducible overexpression, a form of genetic intervention developed by Dr. Mark Kotter of Cambridge University for programming of iPSCs into any cell type, including muscle [91] and fat cells. Meatable’s starting cell type are bovine or porcine umbilical-cord derived stem cells that are then reprogrammed into iPSCs which are further directly converted by forced expression of transcription factors i.e., OPTi-OX technology into desired cell types.

### 2.4. Mesenchymal Stem Cells (MSCs) for Myogenic and Adipogenic Differentiation

Another promising candidate for use in CM production is MSCs which, in addition to their roles in muscle formation [92], also have the ability to differentiate into adipocytes [93], myocytes [94,95], endothelial cells (ECs), and fibroblasts [96]. During muscle formation, most MSCs are involved in myogenic differentiation, producing myofibers and increasing the pool of SCs.

One of the limitations of using MSCs is that MSCs undergo aging in vitro, which can be overcome under suitable culture conditions [97]. On the other hand, MSCs are relatively easy to isolate, and there are various published protocols for MSCs isolation, characterization, and proliferation. In order for these cells to be successfully isolated from different species and tissues, it is necessary to use cell markers that are species- and tissue-specifically expressed by MSCs [98].

#### 2.4.1. Mammalian MSCs for Myogenic Differentiation

MSCs derived from various bovine tissues (umbilical cord, adipose tissue, amniotic fluid, endometrium, bone marrow, etc.) have been shown to express mesenchymal markers such as CD105, CD166, CD29, CD73, CD44, and CD90 [99,100], as well as pluripotency markers such as SOX2, NANOG and OCT4 [100], which supports the idea that MSCs may have the ability to even be pluripotent and to differentiate into three germ layers [98]. However, most applications still regard MSCs as multipotent i.e., possessing the ability to undergo main types of mesenchymal differentiation: adipogenesis, myogenesis, chondrogenesis, and osteogenesis [101].

Regarding equine species, MSCs derived from three different tissues (bone marrow, adipose tissue, umbilical cord) have been shown to express the same cell markers—CD44, CD90, and CD105 [98]. Isolated MSCs can be induced and differentiated into myofibers, implying their use as a starting cell type for initiation of CM production. In this regard, a study conducted by Okamura et al. presents three protocols for in vitro myogenic differentiation of MSCs derived from fetal bovine bone marrow based on the use of DNA methyltransferase inhibitor 5-Aza-2′-deoxycytidine (5-Aza) and myoblast-secreted factor Galectin-1 (Gal-1), as well as SkGM-2 BulletKit myoblast culture medium [95]. Also, Ramírez-Espinosa et al. first successfully induced differentiation of bovine (*Bos taurus*) bone marrow-derived MSCs (BM-MSCs) into myogenic or adipogenic lineages, and then evaluated the effect of peroxisome proliferator-activated receptor-gamma (PPARγ) agonists on the differentiation and metabolic characteristics of these cells [94].

#### 2.4.2. Avian MSCs for Myogenic Differentiation

Chicken MSCs can be used as an avian culture model to learn more about myogenic, adipogenic, and osteogenic pathways. The availability of poultry MSCs-specific markers is limited, so scientists use reports of mammalian cell markers such as CD105, CD90, and CD73, as well as transcription factors that include OCT4, NANOG, and SOX2, where PouV represents a chicken homolog of mammalian OCT4 [102]. Still, chicken MSCs have been successfully derived from a variety of sources such as bone marrow (BM) and compact bones [103], lung, and Wharton’s jelly [104]. Chicken BM-MSCs have properties similar to mammalian MSCs. On the other hand, although the myogenic differentiation of chicken MSCs is still poorly understood, recently Zhou et al. showed that chicken MSCs have the potential for myogenic differentiation by the treatment of dexamethasone (DEX), HC and horse serum, or 5-Aza and horse serum [105].

#### 2.4.3. Mammalian MSCs for Adipogenic Differentiation

In addition to the ability of MSCs to differentiate into the myogenic lineage, these cells can also commit to the adipogenic lineage. Differentiation of MSCs into adipocytes is mainly achieved using a mixture of insulin, isobutylmethylxanthine (IBMX), and DEX, with the PPARγ agonist (e.g., rosiglitazone) frequently included as an additional component [106]. The toxicity of IBMX and DEX’s steroid nature makes this approach problematic for the production of adipose tissue that would be used in CM bioprocess.

When it comes to mammalian adipogenic MSCs, it has been proven that three sources of fetal sheep MSCs (bone marrow, adipose tissue, and liver) have the adipogenic differentiation potential [107].

#### 2.4.4. Fish MSCs for Adipogenic Differentiation

In 2019, a study done by Riera-Heredia et al. demonstrated that fatty acids from fish oil (eicosapentaenoic (EPA) and docosahexaenoic (DHA) acids) and vegetable oils (linoleic (LA) and alpha-linolenic (ALA) acids) have adipogenic potential i.e., promote differentiation of gilthead seabream (*S. aurata*) bone-derived MSCs toward the adipogenic lineage [108].

#### 2.4.5. Fibro-Adipogenic Progenitor Cells (FAPs)

It is important to note that both the traditional livestock industry and CA-based industry, consider only intramuscular fat (IMF) that provides “marbling” as industrially valuable fat since it plays an important role in improving the palatability and flavor of meat [109]. The other types of fat tissue such as subcutaneous and visceral are considered waste by the traditional meat industry and are not relevant for CA-based ones as well. Hence, it is of high importance to consider the cell type from which IMF is predominantly derived, namely **mesenchymal fibro-adipogenic progenitors (FAPs)**, that reside in muscle tissue and have been isolated from various species, including bovine [110].

Interestingly, FAPs have been implicated in post-muscle injury myogenesis [111], particularly the FAP subset expressing glioma-associated oncogene homolog—*Gli1*. Such Gli1+ FAPs are less likely to differentiate into muscle adipocytes but instead participate in enhancing myogenesis and reducing adipogenesis after injury [112].

However, for CA purposes, the pro-adipogenic FAP subsets are of the highest relevance, as well as the procedures for their isolation, proliferation, and adipogenic differentiation.

**Figure 2:**
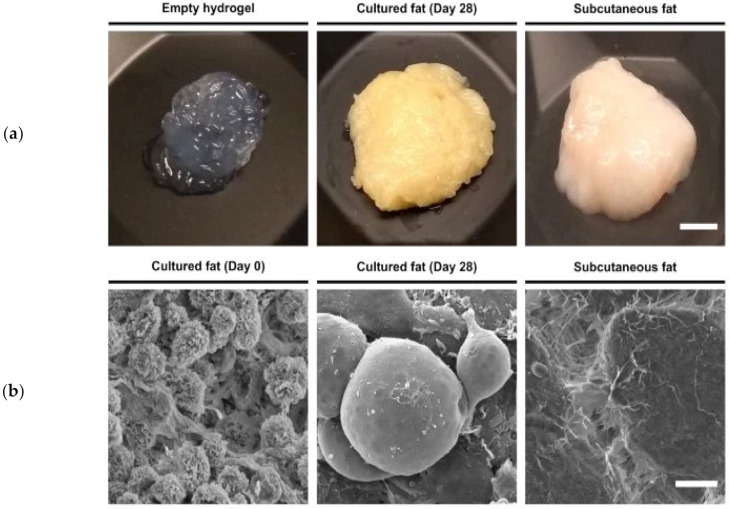
FAP-derived cultured fat in comparison to native bovine subcutaneous fat. (**a**) Macroscopic photographs of empty alginate hydrogel, cultured fat (after 28 days of differentiation), and bovine subcutaneous fat. Scale bar  =  5 mm. (**b**) SEM images of cultured fat after 0 and 28 days of differentiation, and bovine subcutaneous fat. Scale bar  =  10 μm. Reproduced and modified with permission from [113].

FAPs can be sorted by fluorescence-activated cell sorting (FACS) based on the platelet-derived growth factor receptor A (PDGFRA or CD140a) [113]. As shown by Dohmen et al. isolated FAPs are able to undergo many population doublings and can differentiate into CF that has a high resemblance to traditional fat, concerning the appearance, lipid profile, and taste, as shown in Figure 2 [113]. It is worth mentioning that in CA R&D efforts, subcutaneous fat is still of interest for proof-of-concept studies aiming to recapitulate adipogenesis in vitro.

Interestingly, Contreras et al. report a pre-plating strategy for the isolation and culture of an enriched population of FAPs-like adherent cells i.e., muscle connective tissue fibroblasts that respond to TGF-β signaling (that induces FAP proliferation) and the tyrosine kinase inhibitor Nilotinib (inducer of FAP apoptosis) in the same way as FAPs [114]. However, they did not attempt adipogenic differentiation of such pre-plated fibroblasts.

#### 2.4.6. Dedifferentiated Fat (DFAT) Cells

DFAT cells are fibroblast-like multipotent, proliferative cells derived by ceiling culture method from mature lipid-containing adipocytes (MAs). These cells have recently become of interest for cultivated fat (CF) production.

A study done by Peng et al. provided significant insight and evidence into the biological properties of porcine DFAT cells during long-term culture in vitro, such as high cell viability, efficient proliferative capability, normal chromosomal karyotypes, and the capacity to differentiate into adipocytes, myocytes, and osteoblasts [115].

Due to their high proliferative capacity during long-term culture and great adipogenic potential, porcine DFAT cells are promising candidates for CF production on a large scale.

In addition to porcine, bovine DFAT cells have also been investigated for potential use in CF production. Although these cells possess promising proliferative capacity [116], they do not show the same adipogenic potential as porcine DFAT cells. In this regard, extensive work is being done to overcome the given limitation. For instance, treatment with optimal acetate concentrations has been shown to promote bovine DFAT cells’ adipogenesis, i.e., lipid accumulation [117]. Mechanical stimuli have also been implicated in the process of DFAT generation i.e., dedifferentiation of MAs, which is discussed in more detail in the Section 3.2.1 Physical cues.

A summary of the discussed cell types relevant for CM/CF/CS and the animal species the cells are isolated from is presented in Table 1.

### 2.5. Myoblasts and Adipocytes Co-Cultivation

In order to improve the quality of CM/CS in terms of texture, juiciness, tenderness, flavor, and nutritional value by increasing IMF, it is necessary to co-culture adipocytes and myocytes as essential components of meat. in vitro co-culture methods mimic *in vivo* environments and are used to observe interactions among cells and between different cell lines, such as myogenic and adipogenic. Co-cultivation methods can be divided into two major categories—indirect and direct methods. In the indirect methods, cells are physically separated and communicate only via secretory molecules, while direct methods allow cell-cell interactions between different cell types.

Co-culturing techniques are especially challenging because cells in co-culture systems secrete various metabolites that affect the signaling cascades involved in cell proliferation and differentiation. Choi et al. showed that co-culture of bovine muscle SCs with pre-adipocytes increases *C/EBPβ* and *PPARγ* gene expression in differentiated myoblasts and increases *GPR43* gene expression in adipocytes, demonstrating that myoblasts/adipocytes co-culture increases adipogenic gene expression in the myogenic cells [137]. Moreover, Seo et al. suggest that 3T3-L1 adipocyte-induced IL-6 expression in C2C12 myoblasts suppresses their differentiation in a co-culture system [138].

On the other hand, the results of a study done by Chu et al. have shown that during co-cultivation C2C12 cells inhibit the proliferation and differentiation of 3T3-L1 pre-adipocytes by suppressing *glucocorticoid receptor (GR)* gene expression [139]. A recently published study with Tan sheep cells showed a decrease in the number of lipid droplets in the intramuscular preadipocytes (IMPs) that were in a co-culture system with skeletal muscle SCs [140].

When it comes to proliferation, Yan et al. successfully established a co-culture of porcine preadipocytes and muscle SCs and showed that a co-culture system may facilitate the growth and proliferation of cells, while, on the other hand, the same system inhibited cell differentiation [141].

Using ectopic expression, knockdown, and overexpression of the *actin alpha cardiac muscle 1 (ACTC1)* gene, Li et al. have proven that it promotes the differentiation of bovine preadipocytes and myoblasts in the co-culture system, while also affecting myoblast proliferation [142].

Another evidence of how changes in the cell microenvironment (i.e., the co-cultivation system) affect cellular molecular mechanisms is presented as a result of two studies. In the first study performed by Su et al. neudesin neurotrophic factor (NENF) recombinant protein promotes differentiation of bovine preadipocytes while inhibiting bovine myoblast differentiation when the given cell lines are cultured separately [143]. On the other hand, in the second study done by Li et al., it has been shown that exogenously added NENF recombinant protein had a different effect on both bovine preadipocyte and myoblast differentiation in the co-culture system. Namely, the addition of NENF inhibited the accumulation of lipid droplets in bovine preadipocytes, while the differentiation of bovine myoblasts was not significantly affected [144].

Cui et al. have successfully established a co-culture system of dedifferentiated IMPs and SCs from chicken pectoralis major muscle using a transwell chamber. Subsequently, the results of the co-cultivation process showed that actively proliferative SCs affect IMPs by accelerating their differentiation [145].

Generally speaking, as shown above, great achievements have been made concerning the co-cultivation of myoblasts and adipocytes. However, it needs to be emphasized that co-cultivation of myoblasts and adipocytes needs to be performed under strictly defined conditions, optimizing the proliferation or differentiation in both cell types. It is a challenging task, since, as discussed, the expression of adipogenic genes in myogenic cells may occur in a co-culture environment.

It would be worth investigating if there is a time dependence of the adipogenic/myogenic gene expression which might be then manipulated by changing cultivation duration, or if there is a dose dependence of the signaling molecules’ effects.

Concerning 3D co-culture systems, a recent study on 3D co-cultivation of mouse C2C12 myoblasts with 3T3 preadipocytes encapsulated in cell fibers (cell-laden hydrogel microfibers) by alginate shell showed a proportional increase in the number of C2C12 cells with 3T3 fibers [146]. This suggests that the secretome of 3T3 fibers promoted the survival and proliferation of C2C12 cells.

Another interesting 3D co-culture system has been designed by Jo et al. in the form of microtissue made of C2C12 myoblasts and microfiber-based adipose tissue (3T3 preadipocytes) on a polydimethylsiloxane (PDMS) substrate [147].

It would be very useful in terms of CM production, to attempt the same 3D co-cultivation systems using CM-relevant cells, such as bovine, porcine, or chicken. However, the lack of CM-relevant cell lines is hampering such attempts, as discussed in detail in Section 3.1.

#### Need for Performing in Parallel Myogenesis, Adipogenesis, and Vasculogenesis

Muscle and adipose tissue, which are essential parts of skeletal muscle, have well-developed microvascular networks which provide efficient nutrient exchange and gas diffusion required by cells within a tissue. Also, they are associated with the SC niche, regulate muscle tissue maturation by angiocrine signaling and play a major role in generating adipogenesis-required conditions [148].

One of the important tasks in the TE field is the need for in vitro vasculogenesis to be performed in parallel with myogenesis and adipogenesis, in order to generate functional 3D muscle with IMF. It is known that ECs could form microvascular networks through co-culturing with fibroblasts or MSCs [149]. The study conducted by Ma et al. revealed that bovine stromal vascular cells (SVCs) could be used for promoting adipogenesis and angiogenesis in vitro [150]. In 2017, Kayabolen et al. made progress and successfully incorporated vascular structure into developed adipose tissue through co-culturing with ECs [151].

However, the formation of vascular structures is a time-consuming and complex process which makes it unsuitable for application in CM/CF/CS production. Also, since blood vessels are not a crucial component of meat texture and taste, CM/CF/CS production may be simpler without involving the vascularization process. However, when engineering a sizeable 3D tissue construct, it is necessary to achieve proper transport of oxygen and nutrients as well as the elimination of carbon dioxide and other waste products, in order to enable cellular survival. Instead of engineering challenging vascular structures, a promising alternative is the use of perfusion systems, which mimic the vascularization process [19].

**Figure 3:**
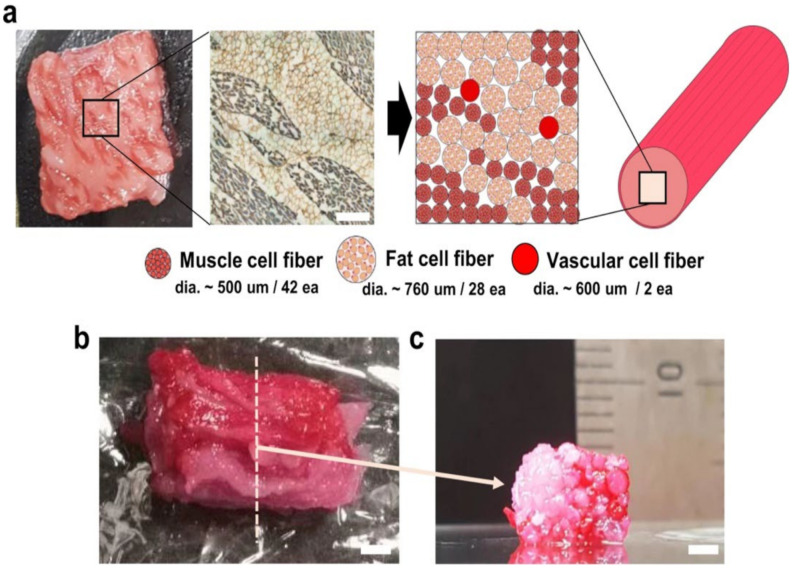
Assembly of fibrous muscle, fat, and vascular tissues to cultured steak. (**a**) Assembly schematic—(right) based sarcomeric α-actinin (blue) and laminin—(brown) stained image (left) of the commercial meat. It is assumed that the diameters of the fibrous muscle, fat, and vascular tissues are about 500, 760, and 600 µm, respectively. Scale bar, 1 mm. (**b**,**c**) Optical images of the cultured steak by assembling muscle, fat and vascular tissues at (**b**) the top and (**c**) cross-section view of the dotted-line area. Muscle and vascular tissue were stained with carmine (red color), but fat tissue was not. Scale bars, 2 mm.—Reproduced with permission from [152].

An interesting study by Kang et al. provides an example of a potential manufacturing protocol for the whole CM product that possesses muscle, fat and vascular components [152]. The authors manually assembled 3D-printed muscle and adipose tissues as well as engineered blood capillaries. The final product resembles a steak as shown in Figure 3.

Dimensions of the product fabricated by Kang et al. (5 mm × 10 mm) are relevant, since the only other reported “whole-cut-like” protocol from the Levenberg group [153], used by Aleph farms company, achieves similar sizes, at least when bioprinting is not used. In the context of co-cultivation effects, it is worth mentioning that the Levenberg group uses multicellular seeding with bovine SCs/bovine smooth muscle cells and/or bovine endothelial cells to achieve higher protein deposition vs. bovine SC monoculture [153].

However, the Kang et al. protocol does not appear very useful for generating larger constructs. It may benefit from switching to the mentioned alternative—placing a 3D printed assembly of muscle and adipose tissues into a perfusion bioreactor.

### 2.6. Stem Cell Normoxia

Another approach that may provide a true insight into cell physiology is to use lower oxygen (O_2_) concentrations (e.g., 1–5%) instead of atmospheric O_2_ concentration (20–21%) in cell and tissue culture experiments. Lower O_2_ concentrations are frequently labeled as “hypoxia”. However, 1–5% O_2_ environment represents “*in situ* normoxia” for most cell types, while atmospheric O_2_ concentration constitutes the hyperoxic state [154].

Several studies show that “*in situ* normoxia” affects muscle SC stemness, i.e., muscle SC proliferation and differentiation. In 2012, Urbani et al. published a study whose results show an increase in the proliferation of mouse SCs under lower O_2_ conditions (2% O_2_) [155]. Recently, Elashry et al. examined the effect of lower O_2_ concentration (3% O_2_) on the regenerative capacity of skeletal muscle-derived SCs in mice, which involved the induction of these cells for myogenic, adipogenic and osteogenic commitments. The results showed that the given O_2_ concentration promotes the SCs multipotency—their myogenesis, adipogenesis, and osteogenesis [156]. The myogenic capacity of porcine SCs is also significantly increased at low O_2_ levels [157].

When it comes to fish cells, a low oxygen environment is a physiological one for these cells, since fish, as aquatic animals, are adapted to tolerate low O_2_ concentrations. For example, there is data on tolerance of low O_2_ for coral reef fish such as goby (*Gobiodon histrio*) and blenny (*Atrosalarias fuscus*) [158].

## 3. Existing Challenges concerning Stem and Progenitor Cells for CA

### 3.1. Need for Immortalized Cell Lines

As CA aims to establish procedures for producing CM/CF/CS and other animal products using TE and synthetic biology, it is crucial to have food-relevant, immortalized cell lines as a starting point of the whole bioprocess. This poses specific challenges to the researchers since the “CA-ready” cells need to be approved as safe for consumption as food and need to have palatable taste, texture, and nutrition characteristics since they will be eventually eaten by the consumers. From the industry point of view, such cells need to be able to undergo efficient proliferation and differentiation at industrial scales. The cell lines should preferentially be stable, homogenous, resistant to environmental fluctuations and ideally, should possess low media requirements [159].

An excellent recent review by Soice and Johnston provides detailed insights into which types of cell lines are most needed for CM and CS research, and eventually production, as well as existing methods of immortalization and their limitations [160].

In this review we provide a summary of the main aspects, as well as the list of valuable CM/CS cell line repositories, most established and maintained by the US non-profit organization The Good Food Institute (GFI) [161,162].

Immortal cells can divide indefinitely. Immortalized cell lines are standard lines that are well characterized, which makes them suitable for further use or manipulation. They are suitable because they allow repeatability of results during cultivation since they represent a genetically identical population. However, the disadvantage is the number of passages that are limited to prevent the accumulation of mutations. Mutations can direct the further course of cell development and function [160].

The use of primary cells is not advantageous in the industry, since even as the living animal tissue biopsy for the cells’ retrieval is a non-invasive process, it is still time-consuming and cost inefficient. In addition, muscle stem cells possess intrinsic limited proliferative capacities which imply the tissue biopsy would need to be repetitive, constantly adding newly sourced cells [163]. Also, animal biopsy cells must be approved for use in food production. On the other hand, immortal cells would not require permanent biopsies precisely because of their ability to divide indefinitely.

When it comes to animal meat and its use in the human diet, skeletal muscle and adipose tissues are the primary targets [164]. Cell lines that would be suitable for the cultivation of these tissues would be SCs, MSCs, fibro/adipogenic stem cells, DFAT cells, and pluripotent stem cells (ESCs and iPSCs) of cows, pigs, chickens, turkeys, and seafood [97]. However, not all the mentioned cell types are equally suitable, due to the difficulties e.g., ease of their isolation (very difficult for e.g., ESCs), sensitivity to culturing conditions, and ease of myo/adipogenic differentiation.

For the muscle tissue, the best choice would be the SCs and myoblasts [165], while for the fat tissue, the MSCs and DFAT cells are the simplest to isolate and culture, and both types can be relatively easily induced to adipogenesis [159]

Currently, for mammalian muscle precursors, there are no commercially available cell lines, and the closest match for R&D-use only are the myoblasts from model species commonly used in other types of research e.g., cancer-related or neuroscience such as mice [166] and rats [167]. Rodent cell lines are not CA-relevant although it needs to be said that particularly mouse C2C12 and 3T3 cells are important “workhorses” in CM/CF R&D studies, as model cell lines.

In the proliferation and expansion phases, most often mediated via MCs for anchor-dependent cells, the cells divide several times to create as many cells as possible. The cells are then transferred to a new environment inducive to differentiation into mature cell types. An obstacle to obtaining a large number of cells in the biological limitation is the number of cell divisions. Cell lines become immortal when they lose the pathways of the cell cycle checkpoints and bypass the aging process. Currently, the following three methods are the most used for establishing immortal cell lines: (1) detection of spontaneously immortal cell lines, (2) expression of the catalytic telomerase subunit (TERT), and (3) induction of viral genes that inactivate p53/p14/Rb. These changes can either occur naturally or be induced by genetic manipulation. The efficient differentiation of iPSC into different cell types is influenced by epigenetic memory, which needs to be taken into account when selecting donor cells from which iPSC lines will be derived. An approach to differentiate human ESCs from skeletal muscle that exploits epigenetic influence [168] suggests the potential application of a similar method for deriving animal skeletal muscle for meat [169].

The patent by Genovese et al. from UPSIDE foods, one of the major CM industry players, on the methods for extending the replicative capacity of somatic cells during an ex vivo cultivation process describes a way to immortalize a cell line using genetic engineering [170]. It builds on the existing method of immortalization of myogenic cell lines by overexpression of reverse transcriptase telomerase (TERT) and cyclin-dependent kinase 4 (CDK4) [171]. In the next patent, on the method for scalable skeletal muscle lineage specification and cultivation, they provide additional innovation by using CRISPR instead of overexpressing CDK4, in order to release a protein that naturally inhibits it [172]. This allows immortalization without the need to ectopically express CDK4.

Spontaneous immortality has certain limitations. On one hand, spontaneously immortalized cells would probably not be considered genetically modified (GM), which could give them access to European markets that currently have strict regulations on GM food. On the other hand, however, spontaneously immortalized cells can be considered equivalent to cancer cells. The process of spontaneous immortalization often leads to a number of additional mutations that are not required for immortalization, and which can change other aspects of cells in unpredictable ways.

In addition, different cell types have different predispositions to spontaneous immortalization. Fish, for example, have a high propensity for spontaneous immortalization due to the naturally high regenerative capacity of their adult stem cell populations [173], while mammals have more regulatory checks to limit spontaneous immortalization.

Consumer studies have shown that taste, diet and safety are important factors for consumers’ readiness for CM and CS [174]. Immortal cell lines used in CM/CS should therefore be developed from cell types and species that are previously known to consumers i.e., that the consumers are accustomed to positively and perceive them as possessing desirable taste and nutritious properties.

One example of the efforts to develop cell lines particularly suitable for CM/CS is the partnership between the GFI and Kerafast which aims to provide a repository of CM/CS-suitable standardized terrestrial and aquatic cell lines [175]. So far, only one cell line deposited in the Kerafast repository has been identified as a candidate for CS—namely the DLEC cell line i.e., continuous adherent cell line derived from early embryos of the European sea bass *Dicentrarchus labrax* [176].

### 3.2. Need for Efficient Stimulation of Differentiation

#### 3.2.1. Physical Cues

In order to complete the process of in vitro myogenesis, it is necessary to induce hypertrophy of the cultured muscle cells since this phenomenon provides maximum protein production, needed in CM/CS products. The hypertrophy stage is achieved *in vivo* via skeletal muscle contractions that promote protein synthesis and myokine secretion [177]. Hence, the in vitro recapitulation needs to enable muscle cell contractions as well.

Such a biomimetic approach stems from the *in vivo* process where activated SCs become proliferative myoblasts through a gene regulation shift involving up-regulation of transcription factors MYF5 and MYOD [178], and down-regulation of PAX7 [179]. These and other regulatory factors with specific spatial and functional restrictions induce the transformation of a myoblast into a myocyte which will then align and fuse with other neighboring myocytes to form multinucleate syncytia i.e., myotubes. This stage is usually recapitulated in vitro by lowering the serum concentration in the medium i.e., by serum starvation. A similar effect can be achieved also by supplementation of ligands for the key surface receptors upregulated during the early phase of myogenesis, such as Insulin-Like Growth Factor 1 Receptor—IGF1R, Transferrin Receptor—TFRC and Lysophosphatidic Acid Receptor 1—LPAR1, as shown by Messmer et al. [180].

In parallel with these genetic changes, the cells *in vivo* undergo structural transformations in order to achieve sarcomeric organization providing contractile functionality realized through the coordinated use of actin, myosin, tropomyosin, calcium, ATP hydrolysis, and other factors.

Although there are plenty of examples of in vitro spontaneous contractions in various cell lines (such as murine C2C12 myoblasts), and iPSC-derived skeletal myocytes, in the majority of primary muscle cell cultures that are still being used by a number of CM/CS companies, spontaneous contractions are not frequently occurring and hence, cells need to be stimulated exogenously by additional mechanical, electrical, or synchronized electromechanical stimuli.

For example, Messmer et al. applied electrical pulse stimulation of serum-free myotubes using a C-PACE EP stimulator at 12 V, 1.0 ms pulse width, and frequencies in the range 0.5–5.0 Hz [181], while Langelaan et al. compared effects of electrical stimulation in 2D monolayers vs. 3D model system, using a 48 h pulsed electrical stimulation protocol, consisting of 4 V/cm, 6 ms pulses at a frequency of 2 Hz [181]. They further compared results obtained for two types of myogenic cells—C2C12 myoblasts as an example of a cell line vs. a primary cell source—muscle progenitor cells (MPCs). They conclude that electrical stimulation, when optimally timed (not sooner than Day 3 of myogenic differentiation), accelerated sarcomere assembly in both 2D and 3D, while there were notable differences in maturation level achieved between different cell sources—with MPC constructs being much more mature than C2C12 constructs, based on developed cross-striations and expression levels of mature myosin heavy chain isoforms [181].

Since skeletal myocytes are excitable cells and their electrophysiological properties are determined by the expression and function of membrane ion channels (sodium, calcium, chloride channels), there are studies investigating transcript levels and expression of these voltage-gated ion channels in physiological and pathological conditions. Currently, this is mostly done within TE research for potential regenerative medicine applications, but it is worth considering if the modulation of ion channel expression and the subsequent impact on skeletal myoblast and skeletal myocyte plasticity may be of use in CM/CS research as well.

As for the physical stimuli that are not directly related to muscle contractions, it is necessary to consider mechanical cues provided by the support structures i.e., scaffolds. Native muscle tissue is an elastic tissue with Young’s modulus of ~10–12 kPa [182,183], hence the scaffolds need to exhibit similar values for mechanical stiffness and elasticity.

One of the most detailed reports up to date on the fabrication of 3D CM constructs by Ben-Arye et al. from the Levenberg group, investigated textured soy protein (TSP) scaffolds seeded with bovine SCs (BSCs) and with co-cultured BSC/bovine smooth muscle cells (BSMC). The mechanical property measurements indicated that both types of constructs (BSC-TSP & BSC/BSMC-TSP) displayed Young’s modulus in the range of the native bovine muscle, but the BSC/BSMC co-culture exhibited higher ultimate tensile strength values and overall similar mechanical properties to native bovine muscle [153].

Kang et al. in their study demonstrating the fabrication of whole-cut cultured meat-like tissue composed of three types of primary bovine cells, report lower values of compressive modulus for the printed constructs vs. native meat, concluding that further optimization of the method is needed [152]. However, the main contribution of this study is still relevant for the challenge to achieve adequate mechanical properties of the CM tissue, since it introduces a modified supporting bath-assisted cell printing method i.e., tendon-gel-integrated printing in which the collagen gel-based tendon tissues can withstand the cell traction force during the bovine SCs differentiation, providing good fibrous structure important for the cell alignment [152].

Concerning adipocytes as the other cell type of major importance for CM bioprocess, the physical stimulation (or purposeful omission of such stimulation) is very relevant in terms of favoring adipogenic differentiation, particularly in connection to the master adipogenic regulator—PPARγ, whose levels of expression are influenced by mechanical loading [184]. We emphasize that only white adipose tissue is of relevance for CM/CF/CS and as such will be discussed here.

In general, it can be said that dynamic or cyclic mechanical strains (stretch or vibrational) suppress adipogenesis, hence the omission of such ways of mechanical loading is inductive to adipogenesis [185].

Li et al. used a dynamic stress cell culture device (Flexcell-5000) to perform mechanical daily stretching for 3 or 5 days on rat bone-marrow isolated MSC culture and detected substantially decreased expression of adipogenic markers: PPARγ-2, adiponectin, and C/EBPα in comparison to the static control group, both in general medium and adipogenic medium [186]. They further show that dynamic stretching upregulated phosphorylation of Smad2 which could be suppressed by pretreatment with the TGFβ1/Smad2 pathway antagonist SB-431542. Such pretreatment was able to reverse the stretch-induced down-expression of adipogenic markers, suggesting that the anti-adipogenic effects of mechanical stretch are, at least to a degree, mediated via the TGFβ1/Smad2 signaling pathway [186].

Other studies further confirm that cyclic loading, in general, inhibits adipogenesis, involving more signaling pathways such as MAPK/ERK that gets activated by uniaxial cyclic stretching as shown by Tanabe et al. using the culture of 3T3-L1 preadipocytes [187]. Stretch-induced activation of the b-catenin signaling pathway is also implicated in the inhibition of adipogenesis of MSCs, as showcased by Sen et al. who used a mouse C3H10T1/2 cell line that displays fibroblastic morphology in cell culture and is considered functionally similar to MSCs [188].

On the other hand, Shoham et al. showed that static mechanical loading stimulates lipid production in 3T3-L1 mouse adipocytes by activating the mitogen-activated protein kinase kinase (MEK) signaling pathway [189]. In this study, they used a custom-build apparatus to apply homogeneous tensile strains to cell culture substrates. The same group of authors further examines these in vitro findings using multiscale modeling, stating a hypothesis that the loading state of the adipocyte plasma membrane (PM) is influenced by neighboring cells, which could imply that adipose cells differentiate as a group, using intercellular positive feedback loops. The authors demonstrate that when the cell density was sufficient (above 19 cells/100 mm^3^), progressive differentiation in some of the adipose cells caused higher magnitudes of tensile strains in the PMs of other nearby cells. This is an interesting hypothesis that needs to be further investigated and confirmed *in vitro,* particularly in 3D culture systems [185].

A study by O’Donnell et al. compared the expression of adipogenic markers (PPARγ-2, adiponectin, leptin, lipoprotein lipase, and perilipin) in monolayer cultures versus both the static and dynamic 3D cultures of human ADSCs made by encapsulating cells into gelatin-based (GelMA) scaffolds. They established, somewhat surprisingly, a decrease in markers’ expression in both types of 3D cultures [190]. The authors hypothesized that the decrease is most likely due to the challenges in the diffusion of pro-adipogenesis factors delivered into the 3D hydrogels and/or potentially related to still unstudied protein–scaffold interactions that may contribute to the sequestration of such factors. The decrease of adipogenic markers in both the static and dynamic 3D cultures is an important preliminary finding that needs to be further investigated, particularly since the fat TE for CM/CF uses is most likely going to be focused around 3D cultures.

When considering DFAT generation through dedifferentiation i.e., reprogramming of MAs, a study by Liu et al. showed that mechanical signals such as substrate stiffness, mechanical stretch, and fluid shear stress can induce such reprogramming through the YAP/TAZ-binding motif [191], which is not surprising since YAP (Yes-associated protein) and transcriptional co-activator with PDZ-binding motif (TAZ) are the main sensors of physical and mechanical forces in the cellular microenvironment [192].

Liu et al. examined the changes in stiffness of the extracellular matrix (ECM) in the human MA culture implementing a so-called Improved Ceiling culture Method that uses significantly reduced volumes of culture media and allows the MAs to gradually adhere to the dish wall and form spindle-like protrusions. When a permeable membrane was introduced on the surface of the culture dish, the dedifferentiation of MAs to DFATs was inhibited, suggesting that a membrane i.e., rigid material as an analog of the rigid ECM plays a role in triggering and initiating reprogramming of MAs. In the group with the membrane, the YAP/TAZ expression levels were significantly reduced in comparison to the normal culture group i.e., without the membrane. Importantly, expression of reprogramming genes such as *Nanog, SOX2, Oct4, and c-Myc* was also significantly reduced in the “membrane group” indicating that ECM stiffness has a major effect on the dedifferentiation process [191].

An interesting observation is that the majority of available research articles dealing with the potential stimulation of adipogenesis and/or dedifferentiation of MAs by manipulating mechanical loading-sensitive signaling pathways are from the research on obesity and related diseases. At the time of writing this review, in April 2022, it was not possible to find an original research article that deals with fat TE for specific use in the CM/CF field, which is why we chose to present available data on the matter, despite the used cell types such as human/rat/mouse adipocytes/MSCs that are not relevant for practical application in CM/CF field but do refer important clues to the loading-dependent signaling pathways of adipogenesis and MAs reprogramming.

It is important to emphasize that there are more parameters to take into account concerning the physicomechanical characterization of the CM/CS engineered tissue constructs—such as total porosity and pore size distribution, liquid uptake, and degradation rate of scaffolds. In the latest publication from the Levenberg group, Ianovici et al. evaluated scaffolds produced by 3D printing with non-animal proteins, namely pea protein isolate (PPI) and soy protein isolate (SPI) with RGD-modified alginate (Alginate (RGD)) and seeded with bovine SCs, for CM fabrication purposes. It is worth mentioning that RGD (Arginine-Glycine-Aspartate) motif is the essential domain for cell adhesion [12]. Ianovici et al. conclude that all formulations are suitable for flexible 3D printing and cell cultivation configuration, and exhibit similar physicomechanical properties except that the pure RGD-alginate underwent the most swelling, had the lowest Young’s modulus, and higher degradation rate, hence one can deduce that incorporation of either SPI or PPI enhances construct stiffness [15].

In the context of mechanical properties and mechanical characterization of engineered CM/CS products, it is worth mentioning a very recent publication by Paredes et al. who presented two methods that can help study CM mechanical characteristics: texture profile analysis (double compression test) and rheology, which can provide data about the elastic and viscous behavior of the samples but also values about other texture characteristics such as springiness, cohesiveness, chewiness, and resilience [193]. All of these parameters are important when evaluating the degree to which CM/CS engineered construct mimics the sensorial properties of already existing commercial products based on traditional meat/fish.

#### 3.2.2. Biochemical Cues

The proliferation and activation of SCs differentiation can be regulated by extracellular signaling molecules (e.g., growth factors—GFs, cytokines, and myokines), which have different biological effects on skeletal muscle function and myogenesis via various signaling pathways, such as Ras/MAPK, JAK/STAT and PI3K/Akt [194]. In this regard, these molecules may have potential roles in CM production. 

Interleukin-6 (IL-6) is an important regulator of myogenesis that promotes proliferation, as well as myogenic differentiation of SCs [195]. In 1997, Quinn and Damon found that interleukin-15 (IL-15) stimulates skeletal myoblast differentiation [196]. In addition to the proven role of leukemia inhibitory factor (LIF) in the proliferation of SCs, the results of a study conducted by Yang et al. demonstrate that LIF induces C2C12 myoblast differentiation through the JAK2/STAT3 signaling pathway—LIF activates STAT3 by inducing its rapid phosphorylation [197]. Also, they confirmed the role of STAT3 in myoblast differentiation by STAT3 knockdown, which significantly blocked myogenesis.

Interleukin-4 (IL-4) has been identified as a signaling molecule with a significant role in myogenesis. Myotubes recruit myoblast fusion by IL-4 secretion, leading to mammalian muscle growth and development [198]. In addition to promoting myogenesis, Chang et al. showed that IL-4 also improves glucose transporter type 4 (GLUT4) translocation and increases glucose uptake by boosting insulin signaling [199].

In 2014, Otis et al. demonstrated for the first time that interleukin-1β (IL-1β) alone can increase the proliferative activity of primary skeletal muscle SCs [200]. Finally, a combination of four pro-inflammatory cytokines secreted by T-cells—interleukin-13 (IL-13), interleukin-1α (IL-1α), interferon-γ (IFN-γ), and tumor necrosis factor-α (TNF-α), has been shown to be able to promote serial SCs proliferation in vitro [201].

Treatment with transforming growth factor-β1 (TGF-β1) has also been shown to improve myogenesis [202].

Lei et al. recently presented an effective four-cytokine combination containing long-chain human IGF-1, PDGF-BB, FGF-2, and epidermal growth factor (EGF) for the expansion of porcine muscle stem cells. They report a 6.31 × 10^7^-fold expansion increase, which renders these results quite industry-relevant. In addition, the same cytokine combination reduced the need for fetal bovine serum (FBS) by at least 5% [194].

When referring to the reduction of FBS used in expansion studies, it is important to explain that one of the main requirements for successful CM/CS commercialization is to reduce and preferably completely omit the use of FBS and other animal-derived components, due to its costs as well as to the inhumane way of its retrieval from unborn bovine fetuses.

FBS, rich in GFs, nutrients, and proteins has been one of the main cell culture media supplements. However, due to its mentioned downsides as well as the insufficient knowledge of the actual components of FBS and batch-to-batch variability in FBS production as well as the potential risk of using serum contaminated by viruses or prions, many CM/CS companies pledged to fully eliminate FBS in their bioprocessing procedures [11].

Hence, when considering an “ideal” culture medium for CM/CS, the main general requirements would be that it is fully chemically defined, free of any animal-derived components, and is affordable to produce. Fulfilling these requirements is not an easy task.

Extensive research has been ongoing in order to define and produce robust xeno-free medium formulations which lead to commercially available media such as Essential 8™, TeSR™, and FBM™ that have enabled the removal of FBS from cell culture. Stout et al. report in a preprint article the application of a modified B8 serum-free media, termed “Beefy-9”, for culturing primary bovine SCs, with short-term growth rates comparable to those obtained in media containing 20% FBS and with the passaged cells maintaining their myogenicity in serum-free conditions [203]. However, the problem is that even though serum-free media stimulate exponential cell expansion, such expansion is lesser than expansion achieved with the growth medium with up to 30% serum [204]. Furthermore, the majority of available serum-free media formulations still contain at least 1 animal-derived component or synthetic mimetics of GFs that would be difficult to get approved for use in food products [205].

Therefore, further advancements are still needed to create chemically defined media formulations that are consistently as effective as serum-based media in promoting cell growth while maintaining targeted differentiation potential e.g., myogenicity.

In addition to serum alternatives, using GFs expressed as recombinant proteins is preferable to animal-derived GFs for similar reasons. Venkatesan et al. in their very recent pre-print article report a set of expression constructs and a simplified protocol for recombinant production of functionally active GFs, including FGF-2, IGF-1, PDGF-BB, and TGF-β1 in *Escherichia coli*. They further use this expression system to produce soluble GFs from different species including bovine, chicken, and fish [206].

On the other hand, besides signaling molecules, a significant increase in myoblast proliferation was observed after co-culture with fibroblasts or macrophages. In triple co-culture, macrophages also continued to promote myoblast proliferation, via biochemical stimulation [207]. However, the authors did not attempt to identify the signaling factors underlying the detected biochemical effect.

When it comes to strictly chemical cues, the study conducted by Fei et al. initially indicated that hydrogen can promote myogenic differentiation of adipose MSCs via the p38 MAPK pathway [208].

When discussing biochemical cues of importance for CM bioprocess, one needs to consider different molecules that regulate fat cell proliferation and differentiation as well.

In their work, Khan et al. confirmed the role of the *Transducer of regulated cAMP response element-binding protein (CREB) 2 (TORC2)* gene in bovine preadipocyte proliferation [209]. In addition, the paper states that four transcription factors (CCAAT/enhancer-binding protein C/BEP, X-box binding protein 1 XBP1, Insulinoma-associated 1 INSM1, and Zinc finger protein 263 ZNF263) have been identified as transcriptional regulators of the *TORC2* gene, which has been confirmed in nuclear extracts of bovine adipocytes via Electrophoretic Mobility Shift Assay [210].

In the study by Yue et al., exosomes derived from bovine adipocytes were isolated and characterized for the first time. mRNA, long non-coding RNA (lncRNA), and microRNA (miRNA) with the potential to regulate the recipient cell phenotype and modulate multiple cell pathways have been identified [211]. The results of this work provide a basis for further studies on the effect of exosomal RNA on adipogenesis.

Long-chain acyl-CoA synthetase 1 (ACSL1) regulates polyunsaturated fatty acids synthesis in bovine adipocytes. In order to improve the nutritional value of beef, it was necessary to investigate the molecular mechanism that uses circular RNAs (circRNA) to regulate ACSL1 and other genes associated with the synthesis of unsaturated fatty acids (UFA). To this aim, Zhao et al. described the RNA-Sec circRNA technique to screen for circRNAs that regulate the *ACSL1* gene expression [212] The same group previously showed that the *ACSL1* gene regulates the UFAs composition in bovine skeletal muscle as well [213].

Overexpression of *CREB-regulated transcription coactivator 3 (CRTC3)* gene has been shown to promote adipogenic differentiation of porcine intramuscular adipocytes by activating the Ca2+-cAMP signaling pathway [214]. On the other hand, Tian et al. suggest that acetyl-CoA acetyltransferase 2 (ACAT2) negatively affects the differentiation of porcine IMPs through the regulation of srebp2/ldlr, cebpα, and PPARγ signaling involved in cholesterol metabolism [215]

A recent study showed that knockdown of Krüppel-like factor 7 (KLF7) inhibits differentiation of goat IMPs, i.e., there is a reduction in the accumulation of lipid droplets, as well as expression of adipogenic markers. On the other hand, Huang et al. showed that fibroblast growth factor 9 (FGF9) inhibits the differentiation of goat intramuscular adipocytes by interacting with the fibroblast growth factor receptor 2 (FGFR2), thus regulating PPARγ and preadipocyte factor 1 (Pref1) [216].

A comparison of goat intramuscular adipocyte and preadipocyte proteomes has revealed many proteins that can potentially play a major role in IMF determination—serine and arginine-rich splicing factor 10 (SRSF10), cysteine and glycine-rich protein 3 (CSRP3), apolipoprotein H (APOH), protein phosphatase 3 regulatory subunit B, alpha (PPP3R1), CREB-regulated transcription coactivator 2 isoform X1 (CRTC2), fructo-oligosaccharides (FOS), plasminogen activator inhibitor 1 (PAI-1/SERPINE1) and allograft inflammatory factor 1 like (AIF1L) [209].

**Figure 4:**
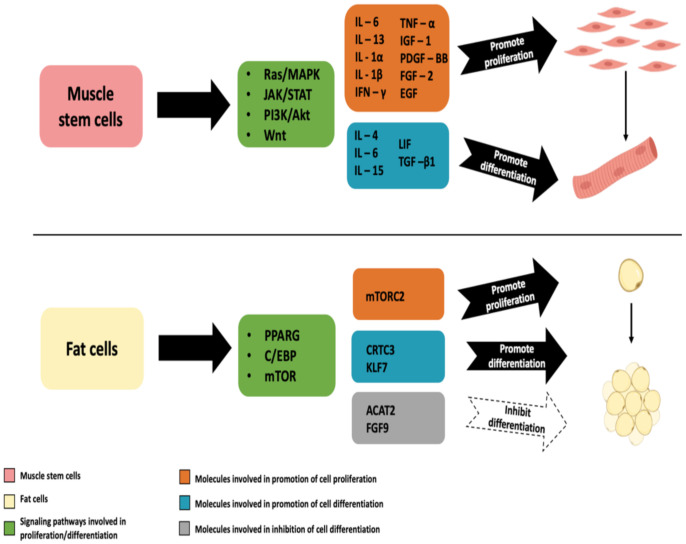
Signaling pathways involved in proliferation and differentiation of myogenic and adipogenic cells.

The focal signaling pathways are summarized in Figure 4.

#### 3.2.3. Using miRNAs as Stimulators of Myoblast Differentiation

Understanding the key actions involved in the process of myogenesis is still one of the main challenges associated with its regulation [217]. The discovery of microRNAs (miRNAs) and their role in the critical regulation of numerous biological processes has provided answers to many questions concerning the modulation of gene expression at the post-transcriptional level. miRNAs represent short and non-coding RNAs that have the ability to regulate genes of interest, usually by specific degradation of mRNA or by translational inhibition [218]. Nowadays it is apparent that miRNA plays a crucial role in almost all aspects of skeletal muscle development.

Like all other complex biological processes, myogenesis is highly regulated by MRFs, whose expression is restricted to the muscle lineage. MRFs participate in the activation of downstream signaling pathways that lead to the formation of muscle fibers.

Well-known MRFs are myogenin, MyoD, Myf5, MRF4 (transcription factors from the MyoD family), and transcription factors from the MEF2 family, as well as serum response factor (SRF) [219]. Considering the significant role of miRNA in muscle gene expression regulation, it was expected that MRFs are directly affected by muscle-specific miRNA targeting influencing the proliferation and differentiation processes. In this review, we emphasize the role of miRNAs in the regulation of myoblast differentiation as summarized in Table 2.

miR-1, miR-206, and miR-133 are some of the best-characterized microRNAs considered to be involved in the regulation of myoblast differentiation. miR-1 affects differentiation by being expressed in skeletal and cardiac muscle cells and can promote muscle cell differentiation by regulation of expression of histone deacetylase 4 (HDAC4) miR-133 which is clustered on the same chromosomal locus as miR-1 has a different molecular mechanism of action comprising repression of serum response factor (SRF). While it is determined that miR-206 has an important role in myogenic differentiation, its underlying mechanism of action is not yet fully identified [220]. However, Jiang et al. showed that *glucose-6-phosphate dehydrogenase (G6PD)* is a novel target gene of miR-206, and further confirmed that miR-206 suppresses muscle cell proliferation by inhibition of *G6PD* expression [221]. An earlier study by Chen et al. indicated that *Pax7* was one of the direct regulatory targets of both miR-1 and miR-206. The authors showed that inhibition of both miR-1 and miR-206 enhances SC proliferation and increases Pax7 protein level *in vivo* [222].

miR-378 is known to be involved in the promotion of skeletal muscle cell differentiation, inducing a reduction of the expression of negative regulators. Namely, its expression is induced at the time of C2C12 differentiation [223].

Another miRNA that promotes skeletal muscle cell differentiation is miR-181. Its targeting gene is gene for homeobox protein Hox-A11, known to be a muscle cell differentiation repressor. The role of Hox-A11 is suppression of expression of *MyoD*, crucial for promoting terminal differentiation. miR-181 targets Hox-A11, suppressing its expression, which leads to the increase of the expression of *MyoD*, promoting muscle cell terminal differentiation [224]. On another hand, miR-374 regulates myoblast differentiation by affecting *Myf6*. Its inhibition by 2′-O-methyl antisense oligonucleotides is proven to increase C2C12 myoblast differentiation, while its overexpression has a negative influence on myogenic differentiation [225].

Besides targeting MRFs, some specific miRNAs are crucial in the manipulation of skeletal muscle cell differentiation. These miRNAs are targeting genes encoding key components of various signaling pathways [217].

For example, in 2020 Huo Lee et al. showed that miR-146b can decrease cell differentiation and promote cell proliferation by regulating the expression of *Platelet-Derived Growth Factor Receptor Beta* (*PDGFRB*). This research showed higher expression of *Inhibitor Of DNA Binding 1* (*ID1)* in pCM cells overexpressing miRNA-146b-5p, which designates that miR-146b-5p can regulate myogenic differentiation indirectly, by regulation of *ID1* [218]. This can be related to the study from 2021 performed by Contreras et al. which shows that changes in *PDGF* family members’ gene expression in murine and human myogenic cells are associated with myogenesis. Specifically, they showed that forced expression of *PDGFRA* can inhibit the myogenesis of skeletal muscle cells while myogenic differentiation reduces the expression of *PDGFRA* [226].

## 4. Achieving Industry Scale CM/CS Production

Ultimately, CM/CF/CS industry aims to reach the large scales of production required for the commercialization of CM/CF and CS products, which implies fulfilling a number of criteria where one of the essential ones is achieving large numbers of cells used as building blocks of CA-based products. When referring to “large-scale” for CM/CS, the volumes of individual bioreactor vessels to consider are comparable to the current industrial microbial fermenters i.e., the volumes should be in the range of 100–1000 m^3^ [227]. The other option is to perform scaling-out or parallelization comprising parallel use of many small-scale vessels. The scaling-out option is more relevant for the differentiation/maturation phase of the CM/CS bioprocess, while scale-up remains the most likely option for the first phase of the CM/CS bioprocess which is proliferation/expansion.

The other key parameters and considerations on scale-up fall outside of the scope of this review and are covered in detail in excellent publications by Chen et al. [228], Bellani et al. [229], Li et al. [227], and Humbird [29].

Concerning the scale-up challenge related to efficient large-scale proliferation i.e., achieving a high number of cells of different types (muscle, fat), there are several aspects to consider. Firstly, one needs to consider that majority of vertebrate cells used in CM/CS are anchorage-dependent (adherent) cells, which implies that for establishing a successful in vitro larger-volume expansion system there are two approaches: (1) to use suspension culture mediated via microcarriers (MCs) that provide the surface for the cells to adhere to or (2) to modify/adapt adherent cells towards anchorage-independent cells [229]

Adaptation of adherent cells towards cells able to proliferate in suspension culture without carriers is difficult and cannot be achieved for all the cell types. Even when such adaptation is successful, additional regular monitoring and dissociation of the cell aggregates is required, as it may otherwise lead to spontaneous differentiation and formation of necrotic cores within aggregates [229]. There are MC-free methods available for muscle cell suspension culture, such as free-floating aggregates of rounded cells—myospheres.

Wei et al. have shown that mammalian SCs have the ability to form free-floating myospheres in suspension after 7–10 days of cultivation. However, prolonged cell cultivation caused loss of the spheroidal shape due to cell damage caused by reduced diffusion of nutrients and oxygen to the center of the myosphere [230].

Therefore, it is recommended to use MCs in the cultivation of muscle cells in suspension, to allow cells to have access to all the components necessary for their undisturbed growth and development.

As for the adipocytes, MCs are not always necessary, since MAs float easily due to high lipid content. However, differentiating adipogenic cells that have not yet accumulated lipid droplets may require MCs to sustain suspension culture—as showcased in the study by Dohmen et al. where bovine FAPs were grown on Cytodex MCs [113].

We refer the readers to the excellent recent review on CF prospects and challenges by Fish et al. [159] for more specifics on the single-cell suspension culture, MC-based culture, or aggregate/spheroid culture types (such as hanging drop technique [150] and method of using adipose spheroids with ECs which can form intraspheroidal vascular-like structures preventing necrotic core formation [231]).

There are different strategies when using MCs, such as using temporary MCs only for the proliferation stage or using non-edible permanent MCs that need to be separated from the cells before going to the next bioprocessing stage. For CM purposes, the ideal scenario would be to use edible MCs that “stay” in the final CM product. Such MCs could also serve as nutrient carriers, providing additional benefits besides anchorage surface. For more details on the MCs for CM bioprocessing, we strongly recommend a review by Bodiou et al. [12].

One of the challenges includes intensification of the cell expansion in MC-mediated suspension culture that can be achieved by taking advantage of the bead-to-bead transfer phenomenon i.e., the ability of already attached cells to transfer and attach to the freshly added MCs.

Hanga et al. discovered that the timing of adding fresh MCs is important (e.g., on Day 5 of culture), as the cells lose the ability for bead-to-bead transfer later in culture (Day 7) when aggregation is high [232]. In addition, Hanga et al. established that a lower starting cell seeding density (1500 cells/cm^2^ for bovine adipose-derived stem cells) is the most cost- and time-efficient, as the lower seeding density enables achieving more doublings in the same bioreactor volume—leading to less processing steps and consequently lower production costs [233].

Another important aspect to consider when planning to scale up is the culture medium as one of the major large-scale bottlenecks since the media-related costs still represent the highest proportion of total costs in CM/CS bioprocessing [19,234]. Different approaches are being applied to reduce the media-related costs including media recycling and modifying the medium composition (covered in Section 3.2.2 Biochemical cues) as well as applying feeding strategies that maximize cell production while minimizing medium consumption. Hanga et al. compared several media exchange options using different volumes of medium in relation to the costs and achieved cell numbers and concluded that the costs of the 80% medium exchange were significantly lower than the costs for 50% medium exchange while yielding 28-fold expansion [233].

## 5. Future Perspectives

A major goal of CA is to achieve an economical and sustainable way of large-scale CM/CF/CS production. However, this goal is at present still not attainable. What are the obstacles to achieving this goal?

Existing challenges can be grouped in several key areas, the majority of which are of scientific and technical nature, but the socio-economic ones are equally important: (1) stable sources of cells; (2) cost-efficient cell culture medium; (3) sustainable and time/cost-efficient large-scale bioprocess design (4) support structures used in different bioprocess phases (MCs and scaffolds) (5) CA-based food safety; (6) consumer adoption & regulatory framework and (7) integration of CM/CF/CS production facilities into conventional meat production ecosystem.

The first challenge of establishing stable sources of cells used as building blocks in CM/CS bioprocessing is the focus of our current review. Such cell lines need not only be stable and safe for human consumption but also usable in industry-scale settings and acceptable to the consumers, who are notoriously against GMO/bio-engineered products. The use of primary cell culture acquired by tissue biopsies is not time- and cost-efficient, as shown throughout the review. Researchers are striving to establish immortalized food-relevant cell lines, a process which has, up to now, been lagging behind other achievements in the APs field.

Besides establishing cell lines, it is also utterly important to devise efficient protocols for the stimulation of proliferation and differentiation of myogenic and adipogenic cells. Major steps have been already done to this aim, as shown in this review, however, there are still many unknowns that need to be resolved.

A very interesting opportunity for gathering more data concerning the differentiation of myogenic cells is ongoing Israel’s Rakia Mission to the International Space Station, launched on 8 April 2022. Within this mission, Israeli leading CM company Aleph farms designed an experiment that investigates the effect of lack of gravity on the cell culture and myogenic differentiation of pluripotent cells. Another aim of the experiment is to take advantage of the limited resources existing in space. Namely, if the experiment shows that it is possible to design and implement a CM bioprocess in such limited resources, this will indicate that a similar CM bioprocess design can be utilized back on Earth within a circular manufacturing process with a reduced carbon footprint.

Besides costs related to the whole CM/CS bioprocess and technical difficulties to establish cell lines that need to be overcome, one must also consider the food safety aspects—since the cell cultures do not possess an immune system and cannot fight off the contamination on their own. Even though the CM/CS bioprocessing is performed under strict sterile conditions, there are still major concerns that contamination can occur, particularly when transferring to industry-scale settings. Currently for R&D phases of CM/CS bioprocess development, low doses of antibiotics are still often used, similarly to other cell culture systems, in order to mitigate bacterial contamination risks. Predictions are that low-dose antibiotics will be necessary also in the production-scale systems in the future as well [235]. However, this is not ideal, particularly in view of the antimicrobial resistance emergence phenomenon. Another issue is the viral contamination, especially concerning since the COVID-19 pandemic of a zoonotic virus. In order to address these issues, it will be necessary to develop advanced sensing tools that can detect potential microbial contaminants such as bacteria and viruses at ultralow levels relevant to industrial needs and devise innovative, non-antibiotic strategies to inhibit both microbial threats without resistance emergence. It is also necessary to devote more efforts to the characterization of other potential risks in CM/CS bioprocessing, as well as to intensify activities aimed at documenting the sensorial and nutritional properties of CM/CS products.

Apart from technical and scientific challenges to CM/CF/CS upscaling and commercialization, very important aspects to consider for future improvements are consumer acceptance and regulatory legislation. As with any type of novel food, regulatory framework and public acceptance of CM/CF/CS are the keys to widespread adoption. Multidisciplinary collaboration between various stakeholders, including scientists, social science researchers, economists, and marketing experts are necessary in order to create social and legal space for acceptance of CM/CF/CS products.

Importantly, members of the public need to be involved in the whole process of introducing CM/CF/CS food to the market, making it a primarily innovation-driven and truly co-creative process, offering direct solutions to the customers’ needs. In order to achieve sustainability as well as efficient adoption, CM/CF/CS research needs to step out of the confinement of the private companies. Existing CM/CS companies should begin publicly sharing more of their findings. Equally important, public institutions should take a more active part in funding and performing CM/CF/CS research. A good recent example is an initiative by the Dutch government that has recently announced a €60 million funding for CM and precision fermentation. This is up to now the largest ever public investment in CA.

Concerning the issue of integrating novel CM/CF/CS production into the conventional meat industry, it is not easy to estimate the most likely scenario. As with other aspects of such a nascent field, this facet of CA development will need to be further developed in the years to come.

## Figures and Tables

**Table 1:** List of cell types relevant for CM/CF/CS and animal species the cells are isolated from.

	Cell Type	CM/CF/CS Relevant Cells	Isolated from
**Pluripotent stem cells**	Embryonic stem cells (ESCs)	Mammalian ESCs	Cow [60,65]; Sheep [66]
		Avian ESCs	Chicken eggs [68]
		Fish ESCs	Medaka fish [70]
	Induced pluripotent stem cells (iPSCs)	Mammalian iPSCs	Horse [80,81]; Pig [82,118]; Cow [60,75,119,120]; Sheep [79]; Goat [77]
		Avian iPSCs	Chicken [85,86]
		Fish iPSCs	Koi fish [88]
**Adult stem cells (ASCs)**	Mesenchymal stem cells (MSCs)	Mammalian MSCs for myogenic differentiation	Cow [100,121] Horse [98]
		Avian MSCs for myogenic differentiation	Chicken [103,104,122,123]
		Mammalian MSCs for adipogenic differentiation	Sheep [107]; Cow [124]
		Fish MSCs for adipogenic differentiation	Gilt-head sea bream [108]
	Adipose tissue-derived stem cells (ADSCs)	Avian ADSCs	Chicken [125]
	Fibro-adipogenic progenitors (FAP)	Mammalian FAP	Cow [93]
	Resident muscle stem cells/muscle satellite cells (SCs)	Mammalian myogenic cells	Cow [34,35,36,37,38] Pig [40,126,127,128] Horse [40,41] Rabbit [129]
		Avian myogenic cells	Chicken [40,46,130]; Duck [40]; Turkey [131,132]
		Fish myogenic cells	Rainbow trout [50] Common carp [51] Atlantic salmon [133] Channel catfish [134] Gilthead sea bream [52,135] Danioninae [52] Goldfish [53]
	Dedifferentiated fat (DFAT) cells	Mammalian DFAT	Cow [117,136]; Pig [115]

**Table 2:** miRNA involved in the regulation of myoblast differentiation.

microRNA	Target Gene(s)	Function
miR-1a	*HDAC4*, *Cx43*, *Pax7*, *c-Met*, *G6PD*	Increased expression upon myoblast differentiation
miR-16-5p	*SESN1*	Represses myoblast differentiation
miR-22	*TGF-bR1*	Promotes myocyte differentiation
miR-23a	*Myh 1*, *2 and 4*	Inhibits myoblast differentiation
miR-24	*SMAD2*	Regulates myogenic differentiation
miR-26	*SMAD1*, *SMAD4*, *and Ezh2*	Promotes differentiation of myoblasts
miR-26a	*Ezh2*	Increased expression upon myoblast differentiation
miR-27b	*Pax3*	Increased expression upon myoblast differentiation
miR-29	*YY1*, *Rybp*	Promotes myoblast differentiation
miR-29b/c	*YY1*, *COL1A1*, *ELN,*	Increased expression upon myoblast differentiation
miR-98	*E2F5*	Represses myoblast differentiation
miR-125b	*IGF-II*	Decreased expression upon myoblast differentiation
miR-133	*SRF*, *nPTB*, *UCP2*	Increased expression upon myoblast differentiation
miR-139	*Wnt1*	Represses differentiation
miR-148a	*ROCK1*	Promotes myoblast differentiation
miR-155	*Mef2a*	Inhibits myoblast differentiation
miR-181	*Hox-A11*	Enhances muscle differentiation
miR-186	*Myog 4*	Inhibits myoblast differentiation
miR-199-3p	*IGF-1*, *mTOR*, *RPS6KA6*	Represses myoblast differentiation
miR-206a	*DNApola*, *Fstl1*, *Utrn*, *Cx43*, *TIMP3*, *Pax7*, *c-Met*, *HDAC4*	Increased expression upon myoblast differentiation
miR-208b/499	*Sox6*, *Purβ*, *Sp3*, *HP-1β*	Increased expression upon myoblast differentiation
miR-214	*Ezh2*, *N-Ras*	Increased expression upon myoblast differentiation
miR-221/222	*p27*	Modulate differentiation and maturation of MSC
miR-322/424	*Cdc25A*	Promotes cell cycle quiescence and differentiation
miR-374	*Myf6*	Represses myoblast differentiation
miR-378a-3p	*HDAC4*	Promotes myoblasts differentiation
miR-431	*SMAD4*	Promotes myoblasts differentiation
miR-486	*FoxO1*, *PTEN*, *Pax7*	Increased expression upon myoblast differentiation
miR-503	*Cdc25A*	Increased expression upon myoblast differentiation

## Data Availability

Not applicable.

## Abbreviations

ACAT2	acetyl-CoA acetyltransferase 2
ACSL1	Long-chain acyl-CoA synthetase 1
ACTC1	actin alpha cardiac muscle 1gene
ADSCs	adipose tissue-derived stem cells
AIF1L	allograft inflammatory factor 1 like
ALA	alpha-linolenic acid
APOH	apolipoprotein H
APs	alternative proteins
ASCs	adult stem cells
ATP	adenosine triphosphate
BM	bone marrow
BM-MSCs	bone marrow-derived mesenchymal stem cells
BSCs	bovine satellite cells
BSMC	bovine smooth muscle cells
CA	cellular agriculture
C/BEP	CCAAT/enhancer-binding protein
CDK4	cyclin-dependent kinase 4
*Cdc25A*	Cell Division Cycle 25A gene
C/EBPα/β	CCAAT enhancer-binding protein alpha/beta
CF	Cultured/cultivated fat
circRNA	Circular RNA
CM	Cultured/cultivated meat
*c-Met*	tyrosine-protein kinase Met gene
*COL1A1*	pro-alpha1 chains of type I collagen coding gene
COVID-19	Coronavirus Disease 2019
CRISPR	clustered regularly interspaced short palindromic repeats
CRTC2	CREB-regulated transcription coactivator 2 isoform X1
CRTC3	CREB-regulated transcription coactivator 3
CS	cultivated seafood
CSRP3	cysteine and glycine-rich protein 3
*Cx43*	connexin 43 gene
DEX	dexamethasone
DFAT	Dedifferentiated fat
DHA	docosahexaenoic acid
DMEM	Dulbecco’s Modified Eagle Medium
DNA	deoxyribonucleic acid
*DNApola*	DNA polymerase alpha gene
DrAMPCs	*Drosophila melanogaster* adult muscle progenitor-like cells
ECM	extracellular matrix
ECs	endothelial cells
EGF	epidermal growth factor
*ELN*	Elastin gene
EPA	eicosapentaenoic acid
ESCs	embryonic stem cells
*Ezh2*	Enhancer Of Zeste 2 Polycomb Repressive Complex 2 Subunit gene
*E2F5*	E2F Transcription Factor 5 gene
FACS	fluorescence-activated cell sorting
FAO	Food and Agriculture Organization of the United Nations
FAPs	fibro-adipogenic progenitor cells
FBS	fetal bovine serum
FFCs	feather follicular cells
FGF-2	fibroblast growth factor-2/basic fibroblast growth factor
FGF9	fibroblast growth factor 9
FGFR2	fibroblast growth factor receptor 2
FOS	fructo-oligosaccharides
*FoxO1*	Forkhead Box O1 gene
*Fstl1*	Follistatin-like 1 gene
Gal-1	Galectin-1
GelMA	Gelatin methacryloyl
GFI	Good Food Institute
GFs	growth factors
GLUT4	glucose transporter type 4
GM	genetically modified
GMO	genetically modified organism
GPR43	G-Protein Coupled Receptor 43
GR	glucocorticoid receptor
*G6PD*	glucose-6-phosphate dehydrogenase gene
HC	hydrocortisone
*HDAC4*	Histone Deacetylase 4 gene
HeLa	Henrietta Lacks
*Hox-A11*	Homeobox A11 gene
*HP-1β*	Heterochromatin Protein 1beta gene
IBMX	isobutylmethylxanthine
ID1	Inhibitor Of DNA Binding 1
IFN-γ	interferon-gamma
*IGF-1/2*	Insulin-Like Growth Factor 1/2 gene
IGF1R	Insulin-Like Growth Factor 1 Receptor
IL-1α/β	interleukin-1 alpha/beta
IL-4/6/13/15	interleukin-4/6/13/15
IMF	intramuscular fat
IMPs	intramuscular preadipocytes
INSM1	Insulinoma-associated 1
iPSCs	induced pluripotent stem cells
IWR1	tankyrase/Wnt inhibitor
KLF4	Krüppel-like factor 4
LA	linoleic acid
LIF	leukemia inhibitory factor
lncRNA	long non-coding RNA
LPAR1	Lysophosphatidic Acid Receptor 1
LR^3^-IGF-1	long-chain human insulin growth factor-1
MAPK	mitogen-activated protein kinase
MAs	mature lipid-containing adipocytes
MC	microcarriers
MEF	mouse embryonic fibroblast
*Mef2a*	Myocyte Enhancer Factor 2A gene
miRNAs	microRNAs
MPCs	muscle progenitor cells
MRF	myogenic regulatory factor
*MRF4*	myogenic regulatory factor 4 gene
MSCs	mesenchymal stem cells
*mTOR*	Mechanistic Target Of Rapamycin Kinase gene
*Myf5*	Myogenic Factor 5 gene
*Myf6*	Myogenic Factor 6 gene
*Myh 1/2/4*	Myosin Heavy Chain 1/2/4 gene
MyoD	myoblast determination protein 1
NASA	National Aeronautics and Space Administration
NENF	neudesin neurotrophic factor
*N-Ras*	Neuroblastoma RAS Viral Oncogene Homolog gene
OCT4	octamer-binding transcription factor 4
OPTi-OX	optimized inducible overexpression
O_2_	oxygen
PAI-1/SERPINE1	plasminogen activator inhibitor 1
*Pax3/7*	Paired Box 3/7 gene
PDGF-BB	platelet-derived growth factor BB
PDGFRA/B	platelet-derived growth factor receptor A/B
PDLIM3/5	PDZ And LIM Domain 3/5
PDMS	polydimethylsiloxane
POU5F1	POU domain, class 5, transcription factor 1
PPARγ	peroxisome proliferator-activated receptor-gamma
PPI	pea protein isolate
PPP3R1	protein phosphatase 3 regulatory subunit B, alpha
Pref1	preadipocyte factor 1
*PTEN*	Phosphatase And Tensin Homolog gene
*Purβ*	Purine Rich Element Binding Protein B
RGD	Arginine-Glycine-Aspartate motif
RNA	ribonucleic acid
*ROCK1*	rho-associated, coiled-coil-containing protein kinase 1 gene
*RPS6KA6*	Ribosomal Protein S6 Kinase A6 gene
*Rybp*	RING1 And YY1 Binding Protein gene
R&D	research and development
SB203580	Adezmapimod, p38 MAPK inhibitor
SCs	satellite cells
SEM	scanning electron microscopy
*SESN1*	Sestrin 1 gene
SFA	Singapore Food Agency
SkGM-2	Skeletal Muscle Cell Growth Medium-2
*SMAD1/2/4*	SMAD Family Member 1/2/4 gene
SOX2	SRY-Box Transcription Factor 2
*Sox6*	SRY-Box Transcription Factor 6 gene
SPI	soy protein isolate
*Sp3*	Sp3 Transcription Factor gene
srebp2/ldlr	KLF7-Krüppel-like factor 7
SRF	serum response factor
SRSF10	serine and arginine-rich splicing factor 10
SSEAs	stage-specific embryonic antigens
SVCs	stromal vascular cells
TAZ	transcriptional co-activator with PDZ-binding motif
TE	tissue engineering
TERT	telomerase reverse transcriptase
TFRC	Transferrin Receptor
*TGF-bR1*	transforming growth factor-beta receptor type 1 gene
TGF-β	transforming growth factor-beta
*TIMP3*	TIMP Metallopeptidase Inhibitor 3 gene
TNF-α	tumor necrosis factor-alpha
TORC2	Transducer of regulated cAMP response element-binding protein (CREB) 2
TSP	textured soy protein
*UCP2*	Uncoupling Protein 2 gene
UFA	unsaturated fatty acid
US	United States
*Utrn*	Utrophin gene
*Wnt1*	Wnt Family Member 1 gene
XBP1	X-box binding protein 1
YAP	Yes-associated protein
*YY1*	Yin Yang 1 gene
ZNF263	Zinc finger protein 263
5-Aza	5-Aza-2′-deoxycytidine
